# Study on the adsorption of Zn(II) and Cu(II) in acid mine drainage by fly ash loaded nano-FeS

**DOI:** 10.1038/s41598-024-58815-z

**Published:** 2024-04-30

**Authors:** Xuying Guo, Honglei Fu, Xinle Gao, Zilong Zhao, Zhiyong Hu

**Affiliations:** 1 College of Civil Engineering, Liaoning Technical University, Fuxin, 123000 Liaoning China; 2 College of Science, Liaoning Technical University, Fuxin, 123000 Liaoning China; 3 College of Mining, Liaoning Technical University, Fuxin, 123000 Liaoning China

**Keywords:** Nano FeS, Fly ash, Load, Acid mine drainage, Adsorption characteristics, Environmental sciences, Chemistry, Energy science and technology

## Abstract

Aiming at the acid mine drainage (AMD) in zinc, copper and other heavy metals treatment difficulties, severe pollution of soil and water environment and other problems. Through the ultrasonic precipitation method, this study prepared fly ash-loaded nano-FeS composites (nFeS-F). The effects of nFeS-F dosage, pH, stirring rate, reaction time and initial concentration of the solution on the adsorption of Zn(II) and Cu(II) were investigated. The data were fitted by Lagergren first and second-order kinetic equations, Internal diffusion equation, Langmuir and Freundlich isotherm models, and combined with SEM, TEM, FTIR, TGA, and XPS assays to reveal the mechanism of nFeS-F adsorption of Zn(II) and Cu(II). The results demonstrated that: The removal of Zn(II) and Cu(II) by nFeS-F could reach 83.36% and 70.40%, respectively (The dosage was 8 g/L, pH was 4, time was 150 min, and concentration was 100 mg/L). The adsorption process, mainly chemical adsorption, conforms to the Lagergren second-order kinetic equation (R^2^ = 0.9952 and 0.9932). The adsorption isotherms have a higher fitting degree with the Langmuir model (R^2^ = 0.9964 and 0.9966), and the adsorption is a monolayer adsorption process. This study can provide a reference for treating heavy metals in acid mine drainage and resource utilization of fly ash.

## Introduction

The pollution of heavy metals in the water environment has become an important and challenging problem in environmental pollution control^1^. Among them, the irrational development and utilization of mineral resources have resulted in the generation of substantial volumes of acid mine drainage (AMD) characterized by low pH levels and the presence of various heavy metals such as iron (Fe), manganese (Mn), lead (Pb), copper (Cu), zinc (Zn), among others^2^. Direct discharge can seriously threaten the soil and water environment and human health^3^. Currently, the commonly used treatment methods include the adsorption method^4,5^, biological method^6,7^, and ion exchange method^8,9^. Nevertheless, these methods still have some limitations in practical application due to the need for additional adsorbents, carbon sources, and long treatment cycles. However, the chemical precipitation method is often used to treat high-concentration acidic copper and zinc wastewater because of its simple operation, economical and efficient^10,11^.

Nano-FeS has the advantages of a large specific surface area and strong reducibility, which is considered an efficient reducing agent for treating wastewater containing copper and zinc^12^. Chen^13^ investigated the effect of nano-FeS sol on the treatment of simulated electroplating wastewater containing Cu(II) by batch experiments, and the results showed that the treated wastewater can meet the requirements of the national electroplating wastewater discharge standard (GB21900-2008). Tu^14^ explored the effect of powder and nano-FeS on the remediation of heavy metal-contaminated soil, and the results showed that the effect of nano-FeS on the remediation of Zn-contaminated soil was better than that of powder FeS, the reason is that nano-FeS has a larger specific surface area. Nevertheless, due to the high surface energy of nano-FeS, direct use is easy to cause oxidative agglomeration; it is necessary to seek stable materials as carriers to enhance the stability of nano-FeS^15^. Sun et al.^16^ developed Al_2_O_3_-supported nano-FeS (FeS/Al_2_O_3_), and the results show that FeS/Al_2_O_3_ can uniformly disperse FeS and inhibit its agglomeration, increasing the stability of FeS. Wang et al.^17^ compared the adsorption capacity of chert-coated nano-FeS before and after the adsorption of As(V) by batch adsorption experiments, and the results showed the maximum adsorption of As(V) by the chert after encapsulation of FeS is 30 times higher than that of the chert. The research group has achieved good results by loading nano-FeS onto lignite in the early stage^18^. However, most of the above carrier materials have the problem of high cost. Therefore, it is necessary to select economically stable carrier materials further.

As a solid waste from coal combustion, fly ash is commonly used in the adsorption treatment of wastewater containing heavy metals because of its large specific surface area and developed pore structure^19,20^. Mohan et al.^21^ investigated the adsorption effect of fly ash on Zn(II) and Cu(II) under different conditions by batch experiments, and the results showed that when fly ash concentration is 2 g/L, the removal rates of Zn(II) and Cu(II) were 42% and 39%, respectively. Liu et al.^22^ prepared tetraethylenepentamine-poly thiocarbamate (TEPAMDT) modified fly ash (MFA), and the results showed that when the concentration of Cu(II) is 100 mg/L, the removal rate of Cu(II) can reach 50% when the concentration of MFA is 412 mg/L. In summary, fly ash as an adsorption material; its unit removal is not high, it has a low adsorption rate, and the conventional modification method has a specific limit on improving its adsorption capacity. Therefore, choosing a reasonable method to treat fly ash is necessary.

Based on this, the present writer prepared fly ash-loaded nano-FeS composites (nFeS-F) through the ultrasonic precipitation method. The nFeS-F not only improves the removal ability of fly ash to Zn(II) and Cu(II) but also solves the problem of easy agglomeration of nano-FeS, provides a surface carrier for nano-FeS, and enhances mechanical strength. Moreover, the effects of nFeS-F dosage, initial pH, stirring rate, reaction time, and initial concentration on Zn(II) adsorption and Cu(II) were investigated. Combined with adsorption kinetics, adsorption isotherms, thermodynamics, SEM, TEM, FTIR, TGA, and XPS, revealed the adsorption mechanism of nFeS-F nFeS-F on Zn(II) and Cu(II). This study can effectively solve the difficult problem of treating heavy metals in acid mine wastewater, and can also broaden the way for the resource utilization of fly ash to achieve the economic and social benefits of treating waste with waste.

## Materials and methods

### Materials

Fly ash: It was taken from Fuxin City, Liaoning Province, China (42.01°N, 121.65°E). After the screening, it was soaked in deionized water thrice and dried at 105 °C for later use. The main chemical composition is shown in Table 1.

**Table 1:** Main compositions of Fuxin fly ash.

Constituent	SiO_2_	TiO_2_	Al_2_O_3_	Fe_2_O_3_	MnO	MgO	CaO	Na_2_O	K_2_O	P_2_O_5_
Fly ash	67.10	0.12	19.74	3.35	0.34	2.87	4.00	1.08	1.30	0.10

Experimental water samples: Based on measured data of acid mine wastewater from a coal mine in Yulin City, Shaanxi Province, China. The pH of the simulated acidic wastewater containing copper and zinc was set to 4 (The usage of 0.1 mol/L HNO_3_ and 0.1 mol/L NaOH adjusted), and the appropriate amount of CuSO_4_·5H_2_O and ZnSO_4_·7H_2_O (the reagents were produced by China Pharmaceutical Group Chemical Reagent Co., Ltd., analytical purity) was dissolved in deionized water to obtain a concentration of 100 mg/L Zn(II) and Cu(II) mixed solution.

### Preparation of nFeS-F

Add 4 g fly ash to 200 mL Na_2_S solution, magnetically stir for 8 h, stand for 5 min, and pour out the Na_2_S solution. Place the conical flask with fly ash particles in the ultrasonic cleaner, config 200 mL of FeSO_4_ solution according to a certain iron-sulfur molar ratio, and add FeSO_4_ solution dropwise to the conical flask at room temperature through a peristaltic pump with a constant flow rate, stir with a mechanical stirrer at 350 rpm, and ultrasonicate at 40 kHz for 10 min. Centrifugation, repeated cleaning with deionized water three times refrigerated sealed spare.

### Characterization and detection

The micro-analysis of the nFeS-F and fly ash before and after adsorption by scanning electron microscope (SEM, SIGMA500, Germany). The nFeS-F image was acquired under transmission electron microscopy (TEM, Themis Z, USA). The functional groups of fly ash and nFeS-F before and after adsorption were identified by spectrophotometer (FTIR, Perkinelmer-1000, Germany) in the 400–4000 cm^−1^ range. The thermogravimetric analysis was done by Netzsch (TGA, STA 449C, Germany). The changes of element and metal valence before and after adsorption were determined by X photoelectron spectrometer (XPS, ESCALAB 250, Britain).

The concentration of Zn(II) and Cu(II) was measured using a Z-2000 flame atomic spectrophotometer (HITACHI), and the pH was measured using the pH-100 pH meter (LICHEN).

### Experimental methods

#### The effect of dosage

At room temperature (298.15 K), nFeS-F with masses of 2 g/L, 4 g/L, 6 g/L, 8 g/L, and 10 g/L were added to 200 mL of Zn(II) and Cu(II) simulated wastewater with an initial concentration of 100 mg/L, respectively (the control groups were all set to add the corresponding grams of fly ash with the particle size of 120–150 mesh to the wastewater). The pH of the simulated water samples was adjusted (pH 4). The samples were taken after stirring at 400 rpm for 150 min. The experiment set up three parallel groups; the mean value was taken as the final value.

The removal rates of Zn(II) and Cu(II) (*η*, %) and adsorption capacity (*q*_*e*_, mg/g) were calculated using Eqs. (1) and (2).

1 $$\eta =\frac{{\text{C}}{0}-{\text{C}}{\text{t}}}{{\text{C}}{0}}\times {100} \%$$

2 $${q}_{e} = \frac{\text{(}{\text{C}}{0}-{\text{Ce)}}\times {\text{V}}}{\text{m}}$$

where: *C*_*0*_ is the initial concentration of the solution, mg/L; *C*_*t*_ is the equilibrium concentration of the solution, mg/L; *C*_*e*_ is the mass concentration of the remaining heavy metal ions in the solution at equilibrium, mg/L; *V* is the volume of the solution, L; *m* is the mass of the adsorbent, g.

#### The effect of initial pH

At room temperature, nFeS-F with a mass of 8 g/L was weighed and added to 200 mL of Zn(II) and Cu(II) simulated wastewater with an initial concentration of 100 mg/L, and the pH of the simulated water samples was adjusted (pH 3, 4, 5, 6, 7), respectively. After stirring at 400 rpm for 150 min, samples were taken and tested. The experiment set up three parallel groups; the mean value was taken as the final value.

#### The effect of stirring rate

At room temperature, nFeS-F with a mass of 8 g/L was taken and added to 200 mL of simulated wastewater with an initial concentration of 100 mg/L of Zn(II) and Cu(II), respectively, and the pH of the simulated water samples was adjusted (pH 4). Samples were taken at 250 rpm, 300 rpm, 350 rpm, 400 rpm, and 450 rpm after stirring for 150 min. Three groups of parallel experiments were set up, and the mean value was taken as the final value.

#### The effect of reaction time

At room temperature, nFeS-F with a mass of 8 g/L was added to 200 mL of Zn(II) and Cu(II) simulated wastewater with an initial concentration of 100 mg/L, and the pH of the simulated water samples was adjusted (pH 4), respectively. After stirring at 400 rpm speed for 5 min, 10 min, 20 min, 30 min, 40 min, 50 min, 60 min, 90 min, 120 min, and 150 min, the samples were sampled and detected. The experiment set up three parallel groups; the mean value was taken as the final value.

#### The effect of initial concentration

At room temperature, nFeS-F with a mass of 8 g/L was weighed and added to 200 mL of simulated wastewater with initial concentrations of 60 mg/L, 100 mg/L, 140 mg/L, 180 mg/L, and 200 mg/L of Zn(II) and Cu(II), respectively, and the pH of the simulated water samples was adjusted (pH 4). After stirring at 400 rpm for 150 min, samples were taken and tested. The experiment set up three parallel groups; the mean value was taken as the final value.

#### Adsorption kinetics

The solution was prepared with a pH of 4 and an initial concentration of Zn(II) and Cu(II) of 100 mg/L, respectively. The nFeS-F and fly ash with a mass of 8 g/L were added to 200 mL simulated wastewater and stirred at 400 rpm for 5 min, 10 min, 20 min, 30 min, 40 min, 50 min, 60 min, 90 min, 120 min, and 150 min at room temperature. After that, the Zn(II) and Cu(II) concentrations were sampled and detected.

The Zn(II) and Cu(II) adsorption data on nFeS-F and fly ash were linearly fitted by Lagergren first-order kinetic equation, Lagergren second-order kinetic equation, and internal diffusion equation^23^.

First-order kinetic Eq. (3)^24^:

3 $${\text{ln(}{\text{q}}_{\text{e}}\text{-q}}_{\text{t}}\text{)} = {\text{ln}}{q}_{\text{e}}-{\text{k}}_{1}{\text{t}}$$

Second-order kinetic Eq. (4)^25^:

4 $$\frac{\text{t}}{{\text{q}}_{\text{t}}}=\frac{1}{{\text{k}}_{2}{{\text{q}}}_{\text{e}}^{2}}+\frac{\text{t}}{{\text{q}}_{\text{e}}}$$

Internal diffusion Eq. (5)^26^:

5 $${\text{q}}_{\text{t}}={\text{k}}_{\text{p}}{{\text{t}}}^{1/2}+ \text{C}$$

where: *q*_*t*_ is the adsorption capacity at time t, mg/g; *q*_*e*_ is the adsorption capacity at equilibrium, mg/g; *K*_*1*_ is the first-order adsorption rate constant, min; *K*_*2*_ is the second-order adsorption rate constant, mg/(g min); *K*_*p*_ is the internal diffusion rate constant, mg/(g·min^1/2^); *C* is a parameter related to the boundary layer, mg/g.

#### Adsorption isotherms

The pH of the prepared solution was 4 and the initial concentrations of Zn(II) and Cu(II) were 60 mg/L, 100 mg/L, 140 mg/L, 180 mg/L, and 200 mg/L, respectively. Adding nFeS-F and fly ash with a mass of 8 g/L to 200 mL of simulated wastewater, stirred at 400 rpm for 150 min at room temperature, detected the concentration of Zn(II) and Cu(II) after sampling.

The Langmuir isotherm model and Freundlich isotherm model were used to linearly fit the adsorption of fly ash and nFeS-F on Zn(II) and Cu(II)^27^.

Langmuir isotherm model^28^ (6):

6 $$\frac{{C}_{e}}{{q}_{e}}=\frac{1}{{q}_{m}{K}_{L}}+\frac{{C}_{e}}{{q}_{m}}$$

Freundlich isotherm model^29^ (7):

7 $${\text{ln}}{q}_{e}={{ln}}{K}_{F}+\frac{1}{n}{{ln}}{C}_{e}$$

where: *C*_*e*_ is the equilibrium concentration, mg/L; *q*_*e*_ is the adsorption capacity at equilibrium, mg/g; *q*_*m*_ is the saturated adsorption capacity, mg/g; *K*_*L*_ is the adsorption constant of Langmuir model; *K*_*F*_ is the adsorption constant of Freundlich model; *n* is the adsorption strength correlation constant.

#### Adsorption thermodynamics

To better understand the thermodynamic characteristics of the reaction process, the primary forces governing the adsorption were elucidated by computing the thermodynamic constants for the adsorption of nFeS-F onto Zn(II) and Cu(II). The relevant thermodynamic equations are shown in (8)–(10).

8 $${K}_{c} = \frac{{q}_{e}}{{C}_{e}}$$

9 $$\Delta \text{G } = -RT{\text{ln}}{K}_{c}$$

10 $$\Delta {\text{G}} = \, \Delta {\text{H}}-T\Delta S$$

where: *K*_*c*_ represents the solid–liquid partition coefficient (L/g); *R* denotes the ideal gas constant, 8.314 × 10^−3^ kJ/(mol K); *T* signifies the absolute temperature in thermodynamics (K); *q*_*e*_ denotes the equilibrium adsorption amount (mg/g); *C*_*e*_ stands for the equilibrium concentration (mg/L); Δ*G* indicates the standard Gibbs free energy (kJ/mol); Δ*H* represents the standard enthalpy change of adsorption (kJ/mol); Δ*S* denotes the standard entropy change of adsorption kJ/(mol K).

#### The effect of coexisting ions and ionic strength

In natural aquatic environments, the presence of Zn(II) and Cu(II) often coincides with other heavy metal ions, potentially influencing their removal by nFeS-F. This investigation delved into the impact of coexisting ions and ionic strength on the removal of Zn(II) and Cu(II), utilizing Cd(II) and Ni(II) as representative species.

### Leaching toxicity of nFeS-F

The leaching toxicity of fly ash and nFeS-F was investigated to assess the safety of these materials in water treatment applications. Heavy metal content in the leachate was determined using a flame atomic spectrophotometer, following the guidelines outlined in the Chinese national standards GB 5085.3–2007 and HJ/T 299–2007^30^.

## Results and discussion

### Characterizations

#### SEM analysis

**Figure 1:**
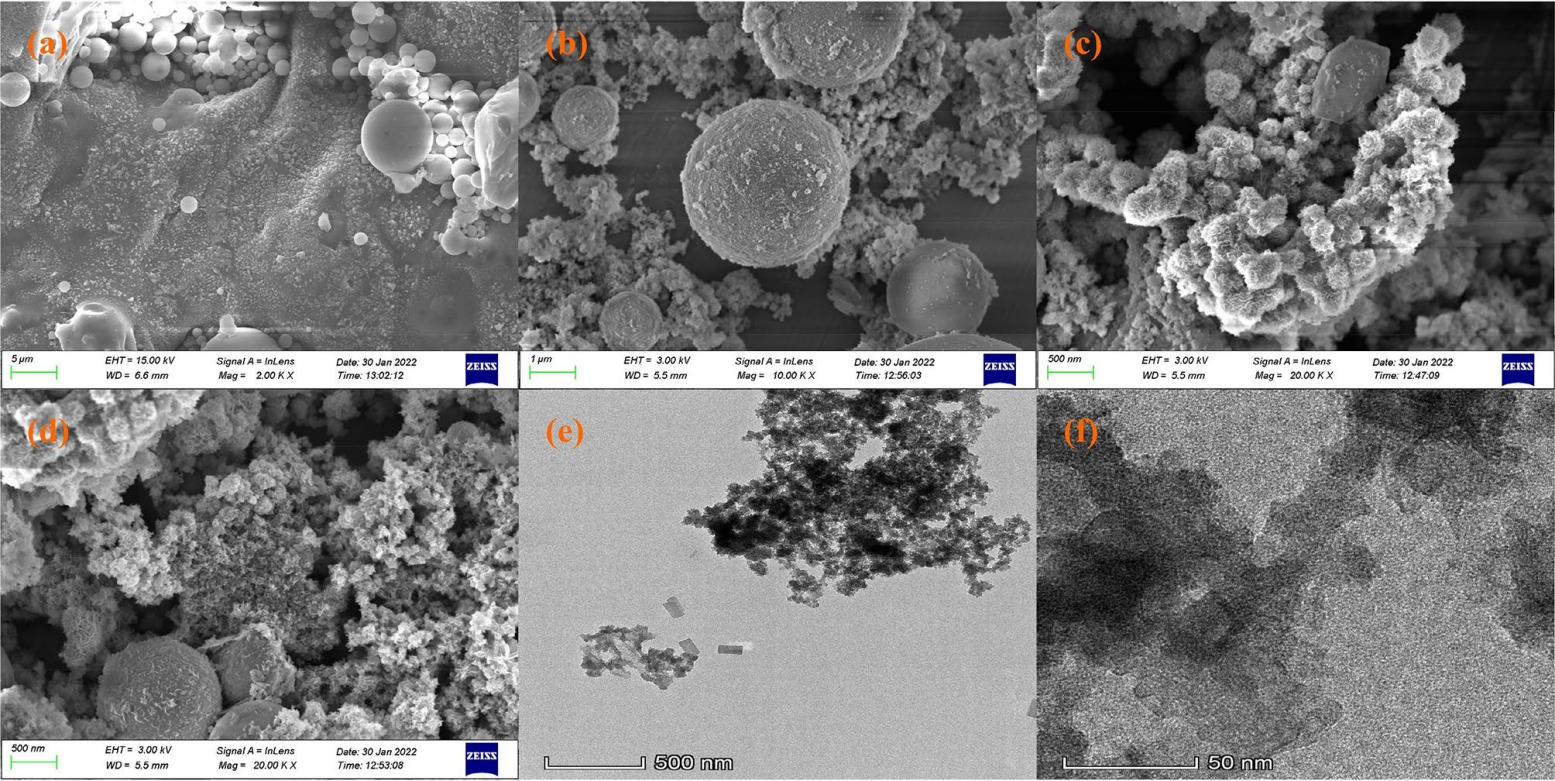
(**a**) Before fly ash adsorption, (**b**) After fly ash adsorption, (**c**) Before nFeS-F adsorption, (**d**) After nFeS-F adsorption, (**e**, **f**)TEM images of nFeS-F.

Figure 1 displays the SEM image of the surface morphology of fly ash and nFeS-F before and after adsorption. As shown in Fig. 1a, fly ash is mainly composed of smooth glassy spheres, irregularly surfaced porous particles, and a small amount of unburned carbon^31^. By comparing Fig. 1a and b, it can be seen that the vitreous structure of fly ash was destroyed after adsorption, the surface roughness increased significantly, and many fine sediments were attached. The sediment crystals may be hydroxide precipitates of Cu(II) and Zn(II). It can be seen from Fig. 1c and d that many flake crystals appear on the surface of fly ash, indicating that nano-FeS is uniformly loaded onto fly ash and the carrier structure is complete. However, after adsorption, the nano-FeS crystals decreased, and the surface of the carrier became rough. At the same time, particle deposition appeared on the surface and pores of nFeS-F particles. The particles should be CuS and ZnS precipitates formed by the reaction of nano-FeS with Cu(II) and Zn(II)^32^.

#### TEM analysis

As depicted in Fig. 1e and f, the FeS crystals exhibit a flaky morphology and are uniformly dispersed across the surface of the fly ash, with an average length ranging from 40 to 80 nm. This morphology bears a resemblance to the nano FeS synthesized by Wang et al.^33^, thereby suggesting, in conjunction with SEM analysis, the efficacy of the ultrasonic precipitation method in loading nano FeS onto fly ash particles. The uniform distribution of nanocrystals on nFeS-F signifies that using fly ash as a carrier can notably enhance the dispersion and stability of nano-FeS, regulate particle size, and effectively mitigate nanoparticle agglomeration.

#### FTIR analysis

**Figure 2:**
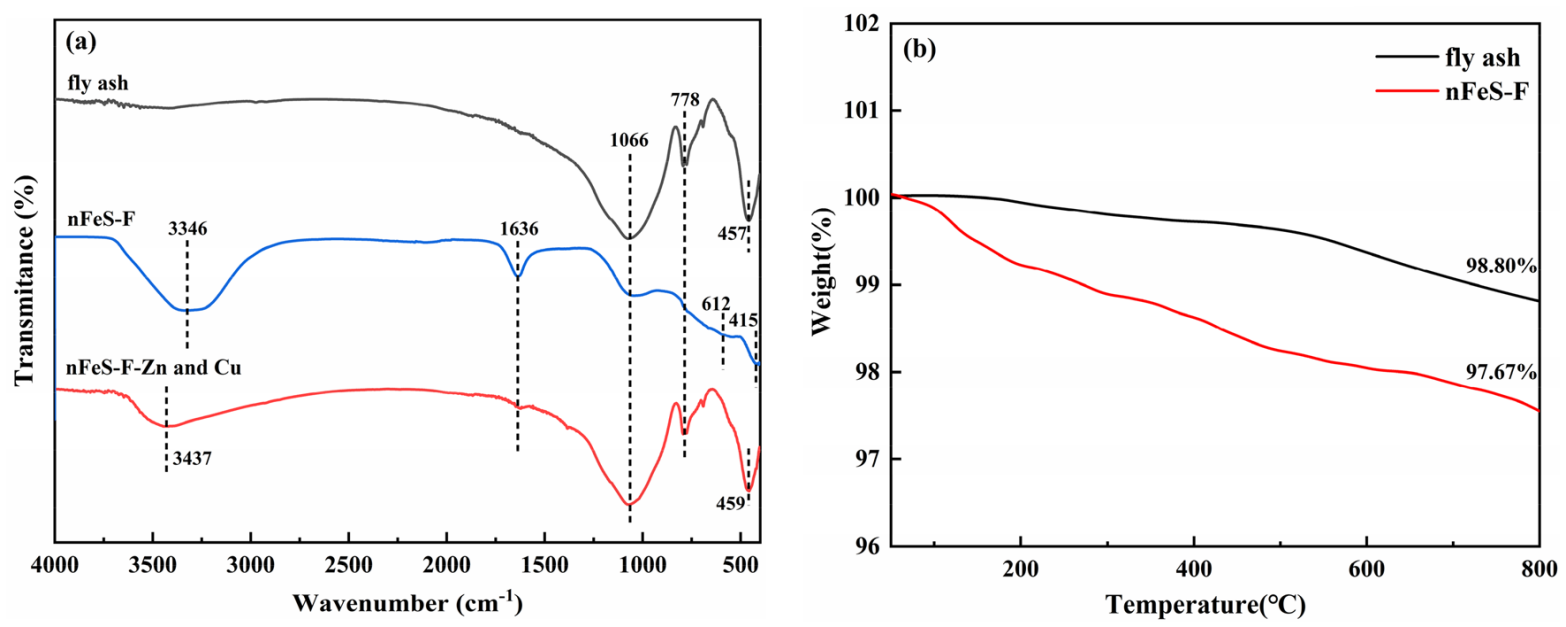
(**a**) The FTIR image of fly ash, nFeS-F before and after adsorption, (**b**) The TGA image of fly ash and nFeS-F.

The FTIR spectra of fly ash before and after adsorption of nFeS-F are shown in Fig. 2a. It can be seen that nFeS-F appears to have a solid and broad absorption peak near 3346 cm^−1^, corresponding to the stretching vibration peak of –OH, indicating that there is a certain amount of hydroxyl group on the surface of nFeS-F^34^. At 1653.89 cm^-1^, H–O–H bending vibration may be the dissipation of bound water on the surface of fly ash. By reducing the adsorption resistance of water film to pollutants, the adsorption of Zn(II) and Cu(II) is enhanced^35^. 1066 cm^−1^, 778 cm^−1^, and 457 cm^–1^ correspond to the asymmetric tensile vibration peak of silica tetrahedron of fly ash, the symmetric stretching vibration peak of Si–O–Si and the bending vibration peak of Si–O, respectively^36^. In previous reports, FeS did not have a prominent absorption peak. In the spectrum of nFeS-F, the bending vibration peak of Si–O moved to the right to 415 cm^−1^, and the intensity of the peak decreased, which may be due to the adhesion of FeS on the surface of fly ash. At the same time, a microstrip of Fe–O groups appeared at 612 cm^−1^, indicating that FeS was oxidized and attached to the surface of nFeS-F through functional groups such as –OH and –C=O^37^. The tensile peak strength of –OH was observed to decrease in the nFeS-F adsorption spectrum, indicating that the hydroxyl group played a certain role in the removal of Zn(II) and Cu(II)^38^.

#### TGA analysis

Thermogravimetric analysis was used to explain the thermal stability and weight loss behavior of fly ash and nFeS-F under a nitrogen atmosphere from 50 °C up to 800 °C. The results of the analysis are shown in Fig. 2b. It was observed that the TGA curve of the fly ash did not change significantly in the tested temperature range, and the total weight loss was only 1.2%, which may be due to the loss of small amount of unburned carbon and moisture in the fly ash, indicating that the fly ash is thermally stable^39^. In contrast, there are two main stages in the weight change mitigation of the thermal decomposition of nFeS-F. Initially, the water on the surface of the material starts to disappear at 110 °C due to the evaporation of the water adsorbed on the surface of nFeS-F, which involves Vander walls force of attraction within molecules^40^. The second weight loss occurred between 200 °C and 480 °C, indicating that FeS underwent oxidative pyrolysis and dissipated as the gaseous products SO_2_ and SO_3_^41^.

#### XPS analysis

**Figure 3:**
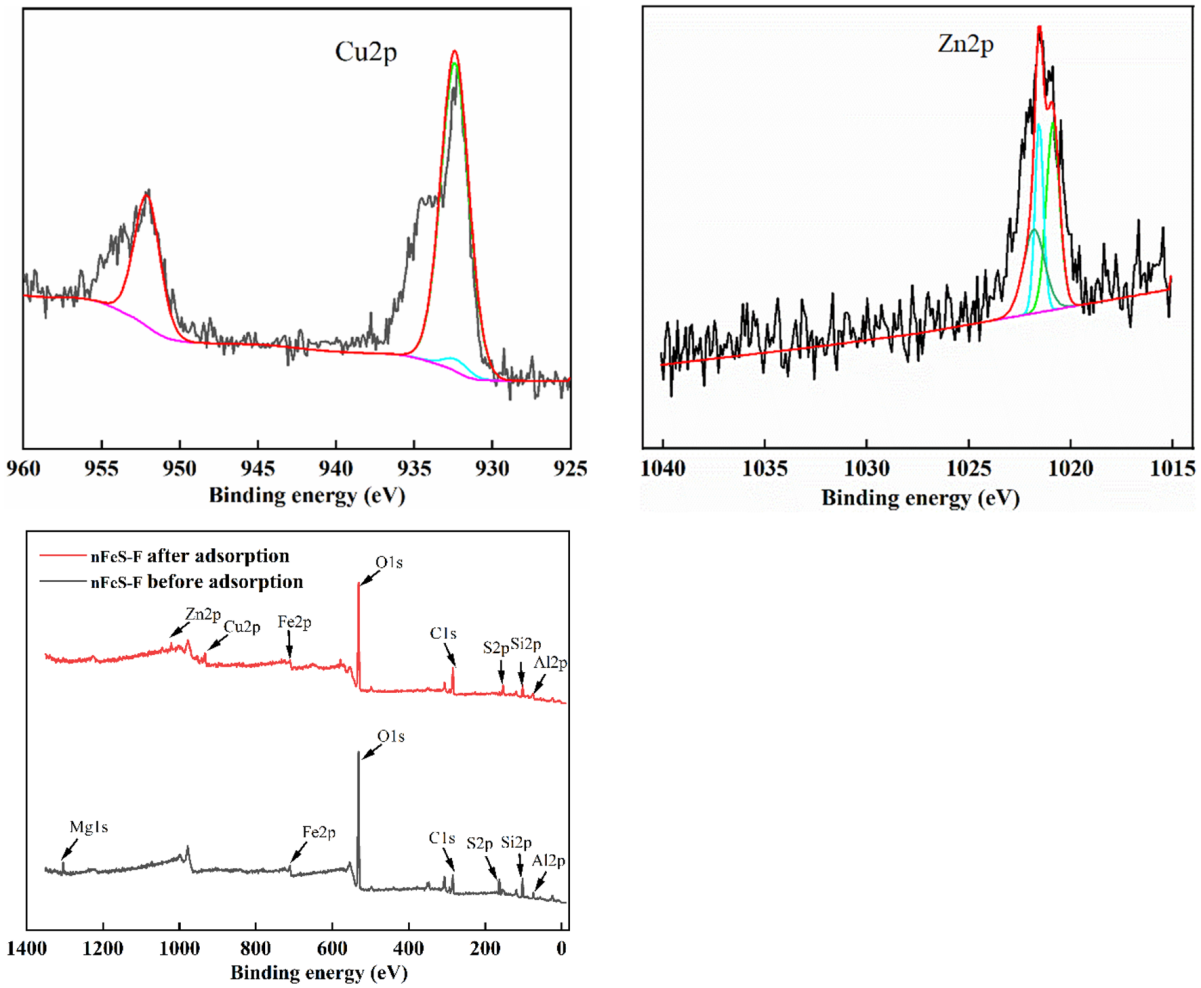
XPS spectra of nFeS-F before and after adsorption.

The changes in elements and valence states before and after the interaction between Zn(II), Cu(II), and nFeS-F were further discussed by XPS analysis. By comparing the changes in the whole spectrum and the relative atomic content before and after the adsorption of Zn(II) and Cu(II) by nFeS-F (Fig. 3, Table 2), the relative atomic content of Fe 2*p* decreased from 3.91% to 2.44%, and the relative atomic content of S 2*p* decreased from 3.31% to 2.23% after nFeS-F treatment of wastewater, indicating that FeS participated in the chemical reaction. At the same time, Zn 2*p* and Cu 2*p* appeared in the adsorbed nFeS-F, and the relative atomic contents were 1.73% and 2.92%, respectively, indicating that nFeS-F had a particular ability to fix zinc and copper. It can be seen that the peak separation diagram of Zn 2*p* corresponds to the characteristic peak of ZnS at 1021.8 eV. The peak of Cu 2*p* corresponds to the characteristic peak of CuS at 932.1 eV^35^, indicating that Cu(II) and Zn(II) were successfully immobilized on the surface of nFeS-F.

**Table 2:** Relative atomic content before and after nFeS-F reaction.

Type	Relative atomic ratio/%								
	C 1*s*	O 1*s*	Al 2*p*	Mg 2*p*	Si 2*p*	Fe 2*p*	S 2*p*	Zn 2*p*	Cu 2*p*
Before nFeS-F reaction	21.57	45.28	7.15	3.15	15.63	3.91	3.31	0	0
After nFeS-F reaction	28.95	44.03	1.58	0	13.01	2.44	2.23	1.73	2.92

### The effect of dosage

**Figure 4:**
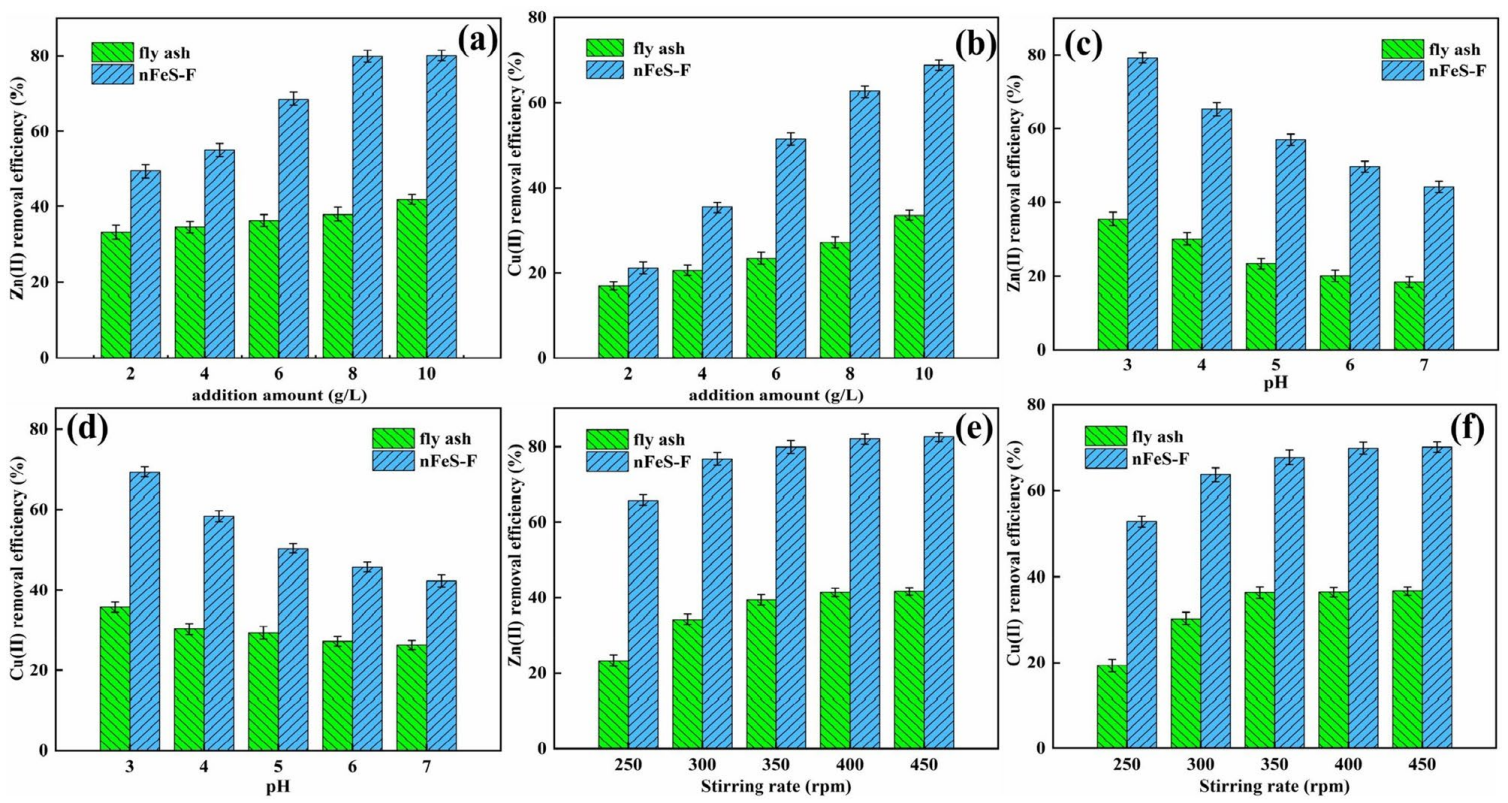
(**a**, **b**) The effect of adsorbent dosage on removal of Zn(II) and Cu(II), (**c**, **d**) The effect of initial pH on removal of Zn(II) and Cu(II), (**e**, **f**) The effect of stirring rate on removal of Zn(II) and Cu(II).

It can be seen from Fig. 4a and b that the removal rates of Zn(II) and Cu(II) increased with the increase of nFeS-F dosage. When the dosage is 2.0 g/L ~ 8.0 g/L, compared with the original fly ash, nFeS-F has apparent advantages, and the removal rate of Zn(II) and Cu(II) increased rapidly, and it increased from 49.50% to 79.92% and from 21.19% to 68.54%, respectively. When the dosage was more than 8.0 g/L, the removal rate of Zn(II) and Cu(II) did not increase significantly, indicating that the adsorption reached saturation when the dosage of nFeS-F was 8.0 g/L, which was the best dosage. Before reaching equilibrium, increasing the dosage of nFeS-F can provide more adsorption sites for Zn(II) and Cu(II) and rapidly increase the removal rate^42^. After reaching equilibrium, the removal effect of nFeS-F is not apparent. The reason is that the concentration of Zn(II) and Cu(II) has been determined, and it is difficult to improve the removal rate even if the dosage of nFeS-F is increased^43^. Therefore, the dosage of nFeS-F in subsequent experiments was set to 8.0 g/L.

### The effect of initial pH

It can be seen from Fig. 4c and d that the removal rates of Zn(II) and Cu(II) decreased gradually with the increase of solution pH. When the pH was 3 ~ 7, the removal rates decreased from 79.21% to 44.36% and from 69.32% to 42.31%, respectively. The reason is that under weak acid conditions, FeS has better solubility and can release more Fe(II) to rapidly reduce Cu(II) and Zn(II)^44^. In addition, Cu(II) and Zn(II) have a smaller solubility product, which makes it easier to react with S(–II) in the solution to form insoluble precipitates such as CuS and ZnS. As the pH continues to increase, the solubility of FeS gradually decreases, and the precipitation of insoluble substances decreases, resulting in a decrease in the removal rate. Considering the situation comprehensively, the pH was set to 4.

### The effect of stirring rate

From Fig. 4e and f, it is evident that the removal efficiencies of both Zn(II) and Cu(II) exhibit an initial increase followed by reaching equilibrium as the stirring rate is intensified. Removing Zn(II) and Cu(II) by nFeS-F demonstrated an escalation from 65.89% to 82.57% and 52.64% to 70.14%, respectively, as the rotational speed elevated from 250 rpm to 450 rpm. This observation suggests that the interaction between nFeS-F and metal molecules is insufficient at lower stirring rates, constraining the solid–liquid phase mass transfer of Zn(II) and Cu(II) on nFeS-F^45^. Stirring rates surpassing 400 rpm yielded marginal increments in Zn(II) and Cu(II) removal efficiencies, indicative of the reaction gradually approaching equilibrium. Consequently, considering all factors, a stirring rate of 400 rpm was chosen for subsequent experiments.

### The effect of reaction time

**Figure 5:**
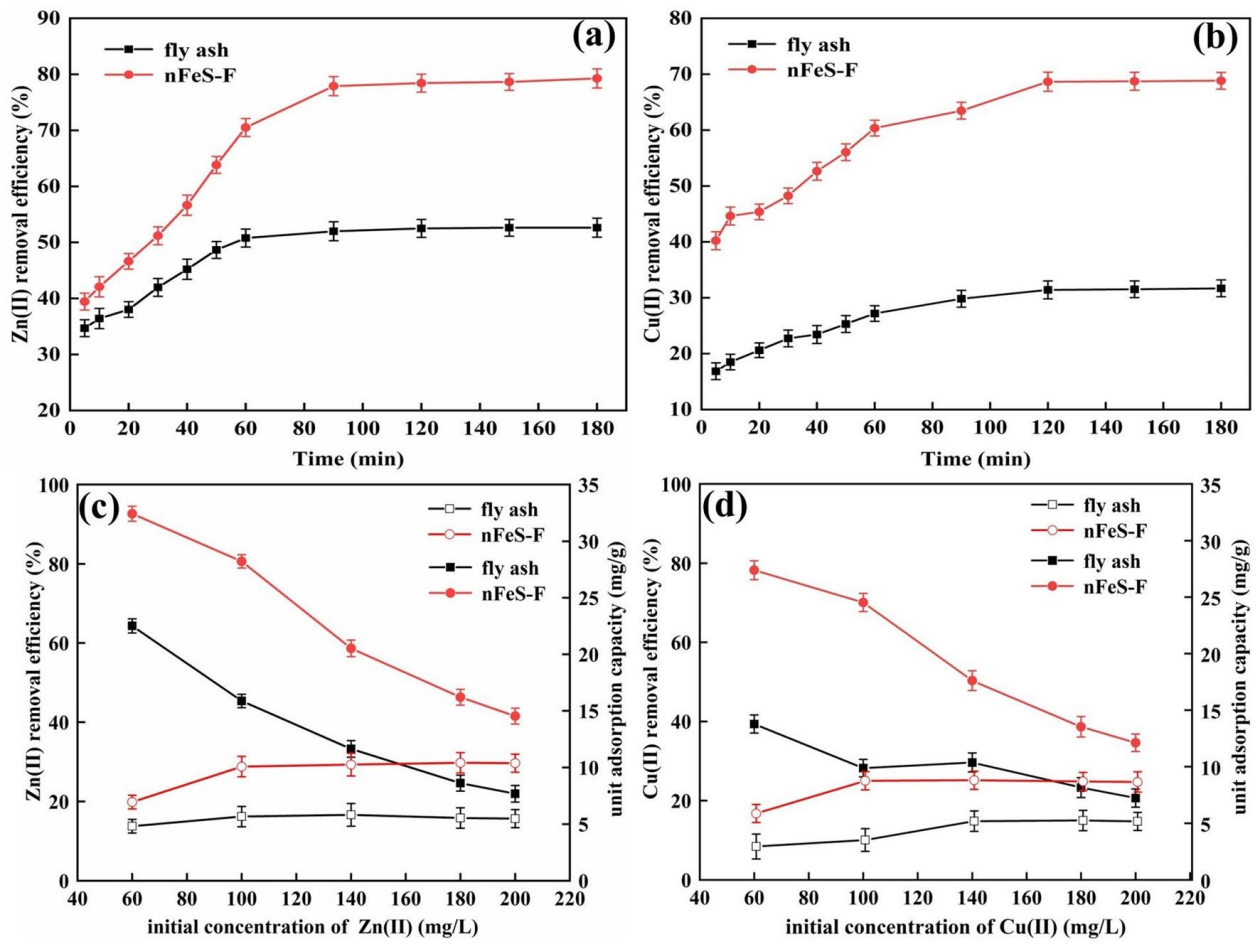
(**a**, **b**)The effect of reaction time on the removal of Zn(II) and Cu(II), (**c**, **d**)The effect of initial concentration on the removal of Zn(II) and Cu(II).

It can be seen in Fig. 5a and b that with the increase of reaction time, the removal rates of Zn(II) and Cu(II) by nFeS-F increased rapidly from 39.45% to 70.51% and 40.21% to 63.47%, respectively. Then, the growth rate gradually decreased (Fig. 3). When carried out the reaction of nFeS-F to Zn(II) and Cu(II) for 90 min and 150 min, the removal rates of Zn(II) and Cu(II) were relatively weak, indicating that the reaction had reached equilibrium. The loaded fly ash can provide more adsorption sites for Zn(II) and Cu(II). At the same time, acidic conditions will promote the rapid dissolution of FeS so that Zn(II) and Cu(II) react with S(–II) to form insoluble sulfides and are removed. When the adsorption time is 0–60 min, Zn(II) and Cu(II) will preferentially enter the surface micropores of nFeS-F. At this time, the concentration of Zn(II) and Cu(II) in the solution is higher, and the adsorption reaction potential kinetic energy is higher, so the removal rate is faster. After the adsorption time continues to increase, the relative content of active sites of nFeS-F gradually decreases, and the mass transfer rate decreases. Considering the situation comprehensively, the reaction time was set at 150 min.

### The effect of initial concentration

It can be seen from Fig. 5c and d that the removal rate of Zn(II) and Cu(II) by nFeS-F is higher when the initial concentration of the solution is low. With the increase of the initial concentration of the solution, the increase of the unit adsorption capacity of nFeS-F to Zn(II) and Cu(II) gradually slowed down, and the removal rate of Zn(II) decreased from 92.69% to 41.54%, when the initial concentration was 60 mg/L to 100 mg/L, the unit adsorption capacity increased from 6.95 mg/g to 10.08 mg/g. When the initial concentration was 100 mg/L to 200 mg/L, the unit adsorption capacity increased slowly. The removal rate of Cu(II) decreased from 78.23% to 34.60%. When the initial concentration was 60 mg/L to 100 mg/L, the unit adsorption capacity increased from 5.87 mg/g to 8.76 mg/g. When the initial concentration was 100 mg/L to 200 mg/L, the unit adsorption capacity increased slowly. Because the initial concentration of Zn(II) and Cu(II) increased, the supply of adsorption sites on the surface of the adsorbent was insufficient, resulting in a decrease in the removal rate. The partial sulfide precipitation formed by the reaction of S(–II) with Zn(II) and Cu(II) blocked the pores of the adsorbent and gradually reached equilibrium^46^. Therefore, the initial concentration of Zn(II) and Cu(II) in the subsequent test was set at 100 mg/L.

### Adsorption kinetic analysis

**Figure 6:**
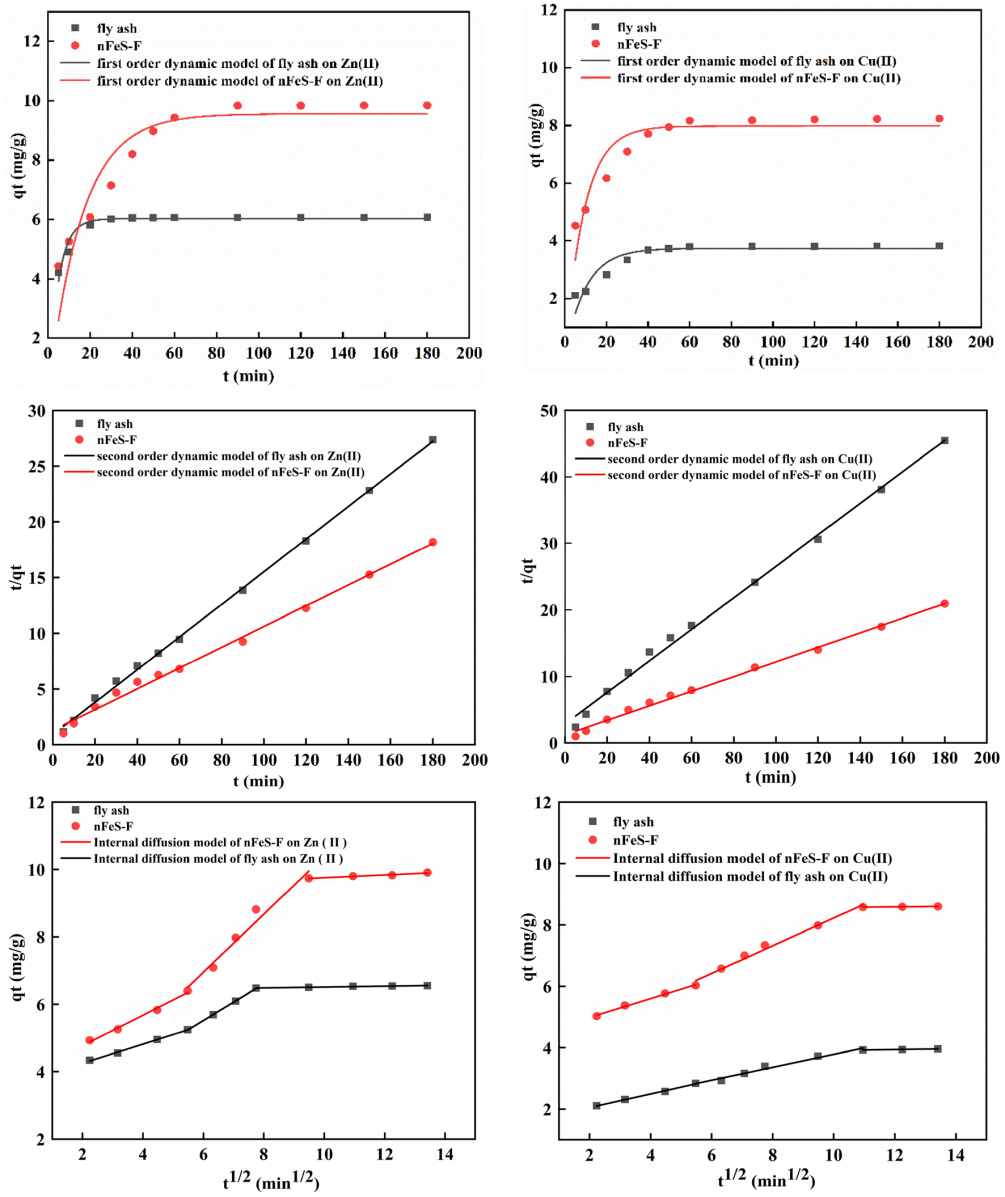
The first-order kinetics, second-order kinetics, and internal diffusion equation of Zn(II) and Cu(II).

The fitting curves and parameters of Zn(II) and Cu(II) are obtained by kinetic equations. The results are shown in Fig. 6 and Table 3.

**Table 3:** The kinetic parameters of Zn(II) and Cu(II) adsorption.

Kinetic equation	Parameter	Cu(II)		Zn(II)	
		nFeS-F	Fly ash	nFeS-F	Fly ash
First-order kinetic equation	*k*_1_	0.1082	0.1010	0.0632	0.2104
	*q*_*e*_	7.9789	3.7364	9.5623	6.0268
	R^2^	0.8277	0.8324	0.8304	0.9252
Second-order kinetic equation	*k*_2_	1.2010 × 10^–2^	5.6041 × 10^–2^	8.6602 × 10^–3^	2.1363 × 10^–2^
	*q*_*e*_	9.1249	4.2242	10.7458	6.8418
	R^2^	0.9952	0.9954	0.9932	0.9987
Internal diffusion equation	*k*_*p*1_	0.3080	0.2229	0.4508	0.2848
	R^2^	0.9911	0.9957	0.9872	0.9961
	C_1_	4.3693	1.6045	3.8753	3.6799
	*k*_*p*2_	0.4515	0.2105	0.8606	0.5411
	R^2^	0.9831	0.9725	0.9586	0.9945
	C_2_	3.7129	1.6800	1.7877	2.2764
	*k*_*p*3_	0.0086	0.0144	0.0409	0.1286
	R^2^	0.9921	0.9533	0.9472	0.9370
	C_3_	8.4866	3.7669	9.3479	6.3807

It can be seen from Table 3 that the correlation coefficient R^2^ of the second-order kinetic equation is higher than that of the first-order kinetic equation, which is 0.9952 and 0.9932, respectively. The calculated theoretical saturated adsorption capacity (q_e_ = 10.7458 mg/g, 9.1249 mg/g) is close to the experimental adsorption capacity (q_m_ = 10.42 mg/g, 8.80 mg/g), indicating that the adsorption of Zn(II) and Cu(II) by nFeS-F conforms to the second-order kinetic equation: t/q_t_ = 0.09306t + 0.9999; t/q_t_ = 0.10959t + 1.0000, adsorption is mainly chemical adsorption process^47^. It can be seen that the internal diffusion equation curve is a continuous multi-segment curve (Fig. 6), indicating that the adsorption of Zn(II) and Cu(II) by nFeS-F is affected by both membrane diffusion and internal diffusion^48^. Zn(II) and Cu(II) adsorption from the liquid phase to nFeS-F includes three stages^49^. The first stage is mainly the initial stage of adsorption. Zn(II) and Cu(II) in the solution diffuse to the surface of nFeS-F and occupy the adsorption sites on the surface of nFeS-F. The second stage is internal diffusion. Zn(II) and Cu(II) enter the pores and are adsorbed after reaching the surface of nFeS-F. The third stage is the adsorption equilibrium stage, and the adsorption gradually reaches saturation.

### Adsorption isotherm analysis

**Figure 7:**
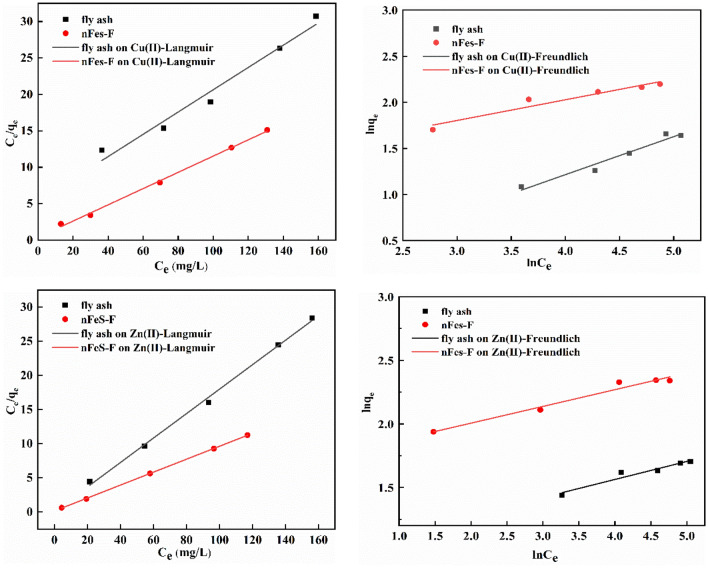
The Cu(II) and Zn(II) adsorption isotherm model fitting.

The adsorption isotherm models were used to fit the experimental data. The obtained adsorption isotherm results are shown in Fig. 7. The fitting equation and specific parameters are shown in Table 4.

**Table 4:** The adsorption isotherm parameters of Zn(II) and Cu(II).

Isotherm model	Parameter	Cu(II)		Zn(II)	
		nFeS-F	Fly ash	nFeS-F	Fly ash
Langmuir isotherm model	*q*_*m*_	2.5898	0.1868	6.4751	11.6670
	*K*_*L*_	3.4639	35.0286	1.6308	0.4786
	R^2^	0.9966	0.9646	0.9998	0.9964
Freundlich isotherm model	*n*	4.45	2.42	7.60	7.06
	*K*_*F*_	3.0943	0.6462	5.7153	2.7113
	R^2^	0.9475	0.9119	0.9268	0.9600

It can be seen in Fig. 7 and Table 4 that the Langmuir fitting curve equations of fly ash and nFeS-F to Cu(II) are as follows: c_e_/q_e_ = 5.35332c_e_ + 0.1528, R^2^ = 0.9646, q_m_ = 0.1868 mg/g; c_e_/q_e_ = 0.38613c_e_ + 0.1114, R^2^ = 0.9966, q_m_ = 2.5898 mg/g. The Freundlich fitting curve equations are lnq_e_ = 0.41322lnc_e_-0.4367, R^2^ = 0.9119; lnq_e_ = 0.22472lnc_e_ + 1.1295, R^2^ = 0.9475; The Langmuir fitting curve equations of fly ash and nFeS-F to Zn(II) are c_e_/q_e_ = 0.08571c_e_ + 0.1790, R^2^ = 0.9964, q_m_ = 11.6670 mg/g; c_e_/q_e_ = 0.15444c_e_ + 0.0947, R^2^ = 0.9998, q_m_ = 6.4751 mg/g. The Freundlich fitting curve equations are lnq_e_ = 0.14164lnc_e_ + 0.9974, R^2^ = 0.9600; lnq_e_ = 0.13158lnc_e_ + 1.7431, R^2^ = 0.9268. Comparing the correlation coefficient R^2^, it can be seen that the Langmuir isotherm fitting of Cu(II) and Zn(II) adsorbed by fly ash and nFeS-F is higher, so the adsorption process is dominated by monolayer adsorption. The adsorption strength n of the Freundlich model is greater than 1, indicating that the adsorption process is easy to carry out^50^. The K_F_ of fly ash and nFeS-F were 0.6462, 2.7113, and 3.0943, 5.7153, respectively. The size of K_F_ was proportional to the adsorption capacity and adsorption strength. By comparison, nFeS-F can effectively improve wastewater-containing Cu(II) and Zn(II) treatment.

### Adsorption thermodynamic analysis

**Figure 8:**
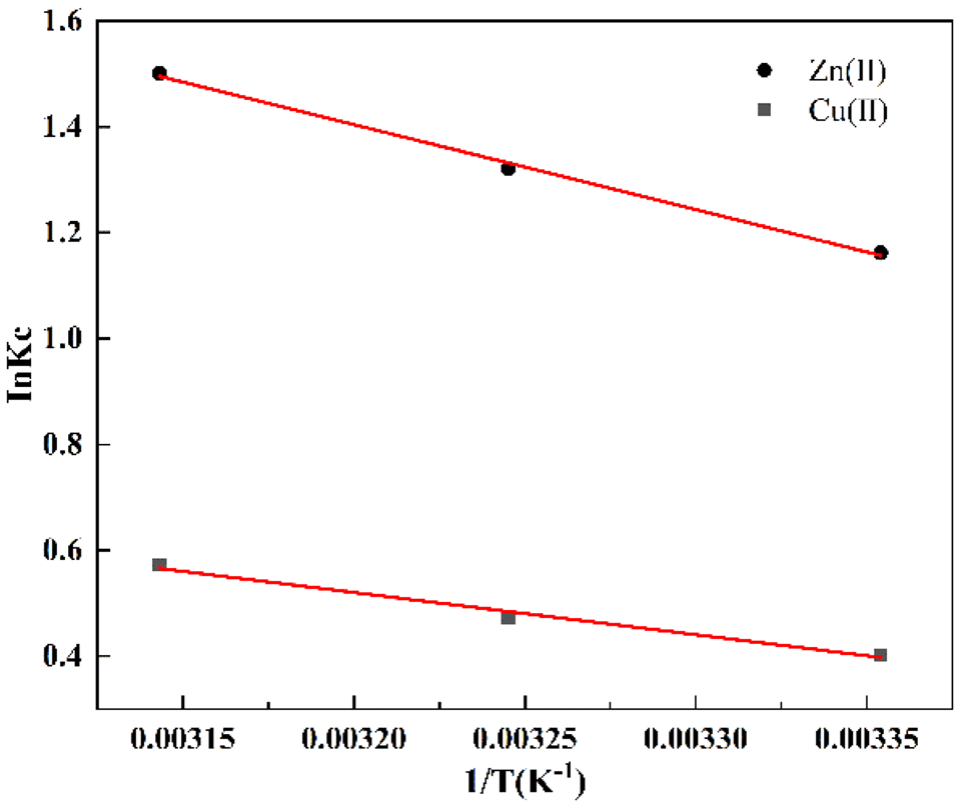
The Van't Hoff plot of 1/T Vs. InKc.

Figure 8 presents the Van't Hoff plots of lnKc against 1/T to determine ∆H and ∆S values. When coupled with Table 5, it becomes evident that the ∆G values for Zn(II) and Cu(II) are consistently negative across all temperatures, diminishing as temperature rises. Conversely, ∆H exhibits a positive trend, suggesting that the adsorption process of nFeS-F onto Zn(II) and Cu(II) is a spontaneous heat-absorption reaction. Furthermore, the temperature elevation proves advantageous for the adsorption of nFeS-F onto Zn(II) and Cu(II)^51^. Positive ΔS values indicate an augmentation in the randomness of the nFeS-F surface, while smaller ΔS values denote a subtle alteration in the adsorbent structure^52^. Table 6 presents a comparative analysis between nFeS-F and other documented adsorbents. When juxtaposed with the adsorbents reported in the literature, nFeS-F exhibits superior Zn(II) and Cu(II) adsorption capacities.

**Table 5:** The thermodynamic parameters of Zn(II) and Cu(II).

	T(K)	∆G(kJ/mol)	ΔH (kJ/mol)	ΔS(kJ/mol K)
Zn(II)	298.15	− 2.8795	1.5979	0.0544
	308.15	− 3.3852		
	318.15	− 3.9697		
Cu(II)	298.15	− 0.9994	6.6335	0.0255
	308.15	− 1.2117		
	318.15	− 1.5124

**Table 6:** The comparison of nFeS-F and other adsorbent materials.

Absorbent	Temperature(K)	*q*_*m*_ (mg/g)		pH	Dosage (g/L)	Equilibrium time (h)	Reference
		Cu(II)	Zn(II)				
nFeS-F	298.15	8.80	10.42	4	8	2.5	This work
Mg Fe-LDHs activated carbon	298.15	2.03		5	2.5	24	^53^
Bentonite supported nano zero-valent iron	278.15	30.96	19.43	3.9	3	2	^54^
ZnCl_2_ activated sludge	298.15	10.56		5	2	24	^55^
Fly ash supported Mg(OH)_2_ nanocomposites	298.15		7.62	2	6	4	^56^
Red earth	300.15		8.74	5.5	24	5	^57^
Manganese oxide-coated sand	298.15		0.12	6	8	1.5	^58^

### The effect of coexisting ions and ionic strength

**Figure 9:**
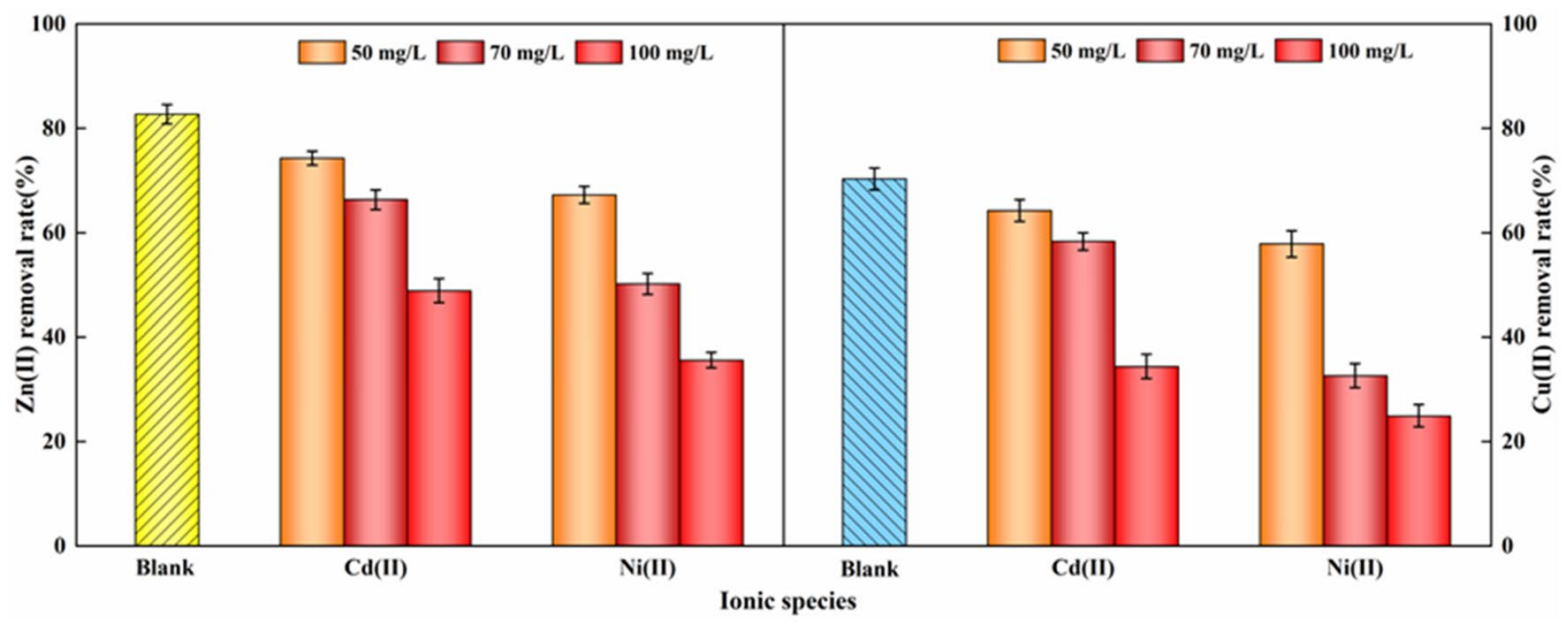
The effect of coexisting ions and ionic strength on the removal rate of Zn(II) and Cu(II) (adsorbent dosage: 8 g/L, solution pH: 4, initial concentration of both Zn(II) and Cu(II): 100 mg/L, reaction temperature: 298.15 K, reaction time: 150 min, stirring speed: 400 rpm).

The analysis of Fig. 9 reveals that coexisting cations hindered the removal efficiency of Zn(II) and Cu(II), with the inhibition effect correlating with the ionic strength. This phenomenon arises from the competitive interaction of coexisting cations with Zn(II) and Cu(II) for adsorption sites, diminishing the electrostatic attraction between nFeS-F and the target ions and reducing removal efficiency. Elevated ionic strength fosters the agglomeration of FeS nanoparticles, thereby diminishing specific surface area and amplifying their inhibitory impact^59^. Furthermore, the competitive adsorption of Ni(II) on Zn(II) and Cu(II) systems surpassed that of Cd(II), likely due to the smaller ionic radius and higher electronegativity of Ni(II)^60^. Consequently, the influence exerted by Cd(II) and Ni(II) on the adsorption of Zn(II) and Cu(II) by nFeS-F ranks as Ni(II) > Cd(II).

### Leaching toxicity of nFeS-F

Table 7 presents the concentrations of main heavy metals in the leach solution of both fly ash and nFeS-F, encompassing total chromium, lead (Pb), copper (Cu), cadmium (Cd), zinc (Zn), and nickel (Ni). Certain heavy metal levels in the fly ash leachate surpassed corresponding regulatory thresholds. Conversely, concentrations of primary heavy metals in the nFeS-F leachate remained below the limits stipulated for direct discharge of water pollutants (as per GB25467-2010^61^) and the standard values for assessing leaching toxicity (GB 5085.3-2007^62^). Suggests that nFeS-F exhibits a commendable ability to immobilize heavy metal ions. Furthermore, the lower concentrations of primary heavy metals in the nFeS-F leach solution than fly ash imply its reduced potential for causing secondary pollution in water treatment scenarios. Thus, nFeS-F is a promising adsorbent for effectively treating wastewater containing Zn(II) and Cu(II).

**Table 7:** The contents of the main heavy metals in fly ash and nFeS-F leachate (mg/L).

Element	Average		Limits for discharged water pollutants (direct emission)	Leaching toxicity identification standard values
	Fly ash	nFeS-F		
Total chromium	0.447	0.194	–	15
Pb	0.243	0.227	0.2	5
Cu	0.286	0.129	0.2	100
Cd	0.198	0.018	0.02	1
Zn	0.184	0.023	1	100
Ni	0.079	ND	0.5	5

*ND* not detected.

## Conclusion

This study synthesized fly ash-loaded nano-FeS composites using the ultrasonic precipitation method based on mineral loading technology. This approach addressed the challenges of the low individual adsorption capacity of fly ash and the susceptibility of nano FeS to oxidative agglomeration, thereby enhancing the stability of the composites. The removal efficiency of nFeS-F for Zn(II) and Cu(II) was investigated through static experiments. Results indicated significantly higher removal efficiency of nFeS-F for Zn(II) and Cu(II) in wastewater than fly ash under acidic conditions, achieving up to 83.36% and 70.40%, respectively. Kinetic and isotherm studies revealed that the adsorption process of Zn(II) and Cu(II) by nFeS-F follows a monomolecular layer adsorption mechanism dominated by chemisorption. Thermodynamic analyses demonstrated that the adsorption of Zn(II) and Cu(II) by nFeS-F is spontaneous. Furthermore, leaching experiments illustrated that nFeS-F significantly mitigated the risk of heavy metal leaching, thus positioning it as an efficient and safe adsorbent for practical applications.

## Data Availability

The datasets used and/or analysed during the current study available from the corresponding author on reasonable request.
